# MALT1-Ubiquitination Triggers Non-Genomic NF-κB/IKK Signaling upon Platelet Activation

**DOI:** 10.1371/journal.pone.0119363

**Published:** 2015-03-06

**Authors:** Zubair A. Karim, Hari Priya Vemana, Fadi T. Khasawneh

**Affiliations:** Department of Pharmaceutical Sciences, College of Pharmacy, Western University of Health Sciences, Pomona, CA, 91766, United States of America; University of Rochester Medical Center, UNITED STATES

## Abstract

We have recently shown that IKK complex plays an important non-genomic role in platelet function, i.e., regulates SNARE machinery-dependent membrane fusion. In this connection, it is well known that MALT1, whose activity is modulated by proteasome, plays an important role in the regulation of IKK complex. Therefore, the present studies investigated the mechanism by which IKK signaling is regulated in the context of the platelet proteasome. It was found that platelets express a functional proteasome, and form CARMA/MALT1/Bcl10 (CBM) complex when activated. Using a pharmacological inhibitor, the proteasome was found to regulate platelet function (aggregation, integrin activation, secretion, phosphatidylserine exposure and changes in intracellular calcium). It was also found to regulate thrombogenesis and physiologic hemostasis. We also observed, upon platelet activation, that MALT1 is ubiquitinated, and this coincides with the activation of the IKK/NF-κB-signaling pathway. Finally, we observed that the proteasome inhibitor blocks CBM complex formation and the interaction of IKKγ and MALT1; abrogates SNARE formation, and the association of MALT1 with TAK1 and TAB2, which are upstream of the CBM complex. Thus, our data demonstrate that MALT1 ubiquitination is critical for the engagement of CBM and IKK complexes, thereby directing platelet signals to the NF-κB pathway.

## Introduction

The proteasome is responsible for selective degradation of damaged proteins and short-lived regulatory proteins, which are crucial in many physiological and pathophysiological cellular processes, including cell cycle progression or inflammation[1–4]. It has been shown to play a major role in nonlysosomal proteolysis in all examined eukaryotic cells[5], and a regulatory role in platelet life span [6–8]. To this end, while it was shown that the proteasome is involved in platelet production, whether it directly regulates platelet signaling and function is still unknown, and warrants investigation. It is to be noted that pharmacological inhibitors of the proteasome have been recently found to influence processes that are known to contribute to the genesis of thrombosis, e.g., they prevent the expression of E-selectin, vascular cell adhesion molecule-1, and P-selectin in activated human endothelial cells[9–11].

While ubiquitination, which is an important post-translational modification, can signal for the degradation of proteins via the proteasome, it has also been established as a mechanism for the regulation of cell signaling[12]. In this connection, we[13] showed that IKK has other roles aside from regulation of transcription[13–15]; specifically, IKKβ, which is a member of the IKK complex, was found to be active in non-nucleated cells, and to play a key role in platelet function (e.g., SNARE machinery-dependent exocytosis).

Moreover, IKKγ, another member of the IKK complex is known to undergo ubiquitination in a number of cell systems, but whether this takes place in platelets remains to be investigated. It is noteworthy that the activity of the IKK complex resides in two catalytic subunits, IKKα (also called IKK 1) and IKKβ (also called IKK 2), and two regulatory subunits, IKKγ(NEMO) and ELKs[16–19]. To this end, given the newly established role of IKKβ in platelet biology, there is increasing interest in understanding and delineating the molecular pathways by which its activity is regulated. Nonetheless, formation of a complex of the CARMA1, Bcl-10 and MALT1 proteins (known as the CBM complex) was found to induce the activation of IKKβ [20–22], albeit in T- and B-cells (hematopoietic origin). Interestingly, while we have previously shown that CARMA1, Bcl-10 and MALT1 proteins are present in platelets, herein we show that they indeed form a complex (i.e., the CBM complex) in these cells. Importantly, one of the members of the CBM complex, namely MALT1 is known to be ubiquitinated in T/B-cells, which is a key step in regulating the CBM complex formation. Specifically, such modification affects the scaffolding function of MALT1 thereby promoting the recruitment of IKKγ[23–25], phosphorylation and degradation of the inhibitory cytoplasmic NF-κB chaperone (IKKα) by the proteasome; which in turn leads[17, 26] to the activation of the IKK complex[23, 24]. However, it is not known whether MALT1 is ubiquitinated in platelets or not. Furthermore, if the platelet MALT1 is indeed ubiquitinated, whether this process regulates IKK activation or not should be investigated. We also believe that understanding the mechanism underlying CBM complex formation and its relationship with IKKβ activity in platelets may define or lead to the discovery of novel antithrombotic targets or agents.

Based on the aforementioned considerations, the present manuscript initially demonstrated the expression of a functional ubiquitin/proteasome system in platelets. We then observed, using a pharmacological inhibitor, that the proteasome regulates platelet function, *in vitro*; namely aggregation, integrin GPIIb-IIIa activation, secretion (dense and alpha granules), phosphatidylserine (PS) exposure, and intracellular calcium changes. Moreover, the proteasome was also found to modulate clot retraction and *in vivo* platelet function (thrombogenesis and hemostasis). Having established the presence of ubiquitin/proteasome system, and identified its physiological role (at least in part) in platelets, we next investigated the ubiquitination of MALT1 and its consequences in the context of IKKβ activation. Our results revealed that MALT1 is in fact ubiquitinated, and that it provides a docking surface for the recruitment of IKKγ to the CBM complex, which would lead to IKKβ activation. In terms of the mechanism, we found that MALT1 is associated with the kinases TAK1 and TAB2 in stimulated platelets, and that they are upstream of CBM complex formation. Finally, our data shows that the proteasome regulates the 7S complex formation of the SNARE machinery, in platelets.

Collectively, our findings support the notion that platelets express a functional proteasome system, and suggest that it may serve as a potential target for managing thrombosis-based disease states.

## Materials and Methods

Human blood studies were approved by the Institutional Review Board (IRB) at Western University of Health Sciences, Pomona, CA, and donors were asked to sign a written consent, and a subjects’ bill of rights, that were previously approved by the IRB.

### Reagents and materials

ADP, MG132 were obtained from Sigma Aldrich (St. Louis, MO). Thrombin, Collagen, stir bars and other disposables were from Chrono-Log (Havertown, PA), and U46619 was obtained from Cayman Chemical (Ann Arbor, MI). The proteasome inhibitor bortezomib (Velcade) was generous gift from Dr. Steven Schwarze (University of Kentucky College of Medicine, Lexington, KY). FITC-conjugated Annexin V, anti—P-selectin, and PAC-1 antibodies were purchased from Cell Signaling Technology, Inc. (Danvers, MA). Antibodies against TAK1, TAB2, CARMA1, MALT1, Bcl10, Ubiquitin, IKKα, IKKβ, IKKγ, VAMP-8, SNAP-23, Syn-11, and pSer/Thr/Tyr from Santa Cruz (Santa Cruz, CA), respectively. Other reagents were of analytical grade.

### Animals

C57BL/6 mice were obtained from Jackson Laboratory (Bar Harbor, ME). Mice were housed in groups of 1–4 at 24°C, under 12/12 light/dark cycles, with access to water and food ad libitum. All experiments involving animals were performed in compliance with the institutional guidelines, and were approved by the Western University of Health Sciences Institutional Animal Care and Use Committee.

### Preparation of platelets

Washed human platelets were prepared as described before[13].

### *In vitro* platelet aggregation

Washed platelets were incubated with MG132 for 5 min and stimulated with thrombin (0.025–0.05 U/mL), or collagen (2.5–5 μg/mL). Platelet aggregation was measured by the turbidometric method using model 700 aggregometry system (Chrono-Log Corporation). Each experiment was repeated at least 3 times, with blood collected from three different human donors.

### ATP release

Platelets were prepared as described above (250 μL; 2.5 × 10^8^/mL) before being placed into siliconized cuvettes and stirred for 5 min at 37°C at 1200 rpm. The luciferase substrate/luciferase mixture (12.5 μL, Chrono-Log) was then added, followed by the addition of the indicated agonists: thrombin and collagen (Chrono-Log).

### Immunoprecipitation

Immunoprecipitation was carried out as described in Karim *et al*. (2013)[13]. Platelets were incubated in the presence or absence of inhibitor and activated with thrombin (0.025 U/mL) followed by lysis with 2× lysis buffer. The lysates were clarified by centrifugation and the supernatants were incubated with rabbit IgG and Protein A-Sepharose 4 Fast Flow (GE Healthcare, Piscataway, NJ). Protein complexes were immunoprecipitated with anti-syntaxin-2, -4, -11, MALT1, CARMA1, Bcl-10, Ubiquitin, IKKα, IKKβ, IKKγ, TAK1 or TAB2 antibodies and Protein A-Sepharose (GE Healthcare). The immunoprecipitates were analyzed by SDS-PAGE and immunoblotting.

### Immunoblotting

Immuno blot was carried out as described before[27].

### Platelet functional responses

Flow cytometric analysis was carried out as discussed in Lin *et al*. (2014)[27]. Briefly, human platelets (2×10^8^) were incubated in the presence or absence of MG132 (10 μM), for 5 minutes and then stimulated with thrombin (0.025 U/mL) for 3 minutes. Platelets were incubated with FITC-conjugated Annexin V, anti—P-selectin, or PAC-1 antibodies at room temperature for 30 min in the dark. Finally, the platelets were diluted 2.5-fold with HEPES/Tyrode buffer (pH 7.4). The samples were transferred to FACS-tubes and fluorescent intensities were measured using a BD Accuri C6 flow cytometer and analyzed using CFlow Plus (BD Biosciences, Franklin Lakes, NJ).

### Fibrin clot retraction assay

With slight modification, the fibrin clot retraction assay was performed as discussed in Osdoit and Rosa[28]. Briefly, whole blood was collected and washed platelets were isolated as discussed above. CaCl_2_ was added extemporaneously at a final concentration of 1 mm. First, a 10% (w/v) polyacrylamide cushion was polymerized at the bottom of the tubes to avoid clot adherence. Tubes were then rinsed extensively in distilled water. Platelets were incubated with MG132 (10 μM) for 5 min. Fibrinogen (500 μg/mL) was added, and clot retraction was initiated by thrombin (0.025 units/mL), at room temperature. Pictures were taken at time intervals using a digital camera, and retraction quantified by digital processing and plotted as percentage of maximal retraction.

### *In vivo* thrombosis model

Mice were IV injected with MG132 (6 mg/kg), bortezomib (2.5 mg/kg) or vehicle and the FeCl_3_-induced thrombosis model was performed after 1 hour as described before[27].

### Tail bleeding time

Mice were IV injected with MG132 (6 mg/kg), bortezomib (2.5 mg/kg) or vehicle and the tail bleeding assay was performed after 1 hour. Hemostasis was examined using the tail transection technique as described before[27].

### Statistical analysis

All experiments were performed at least three times. Analysis of the data was performed using GraphPad PRISM statistical software (San Diego, CA) and presented as mean ± SEM. The Mann-Whitney test was used for the evaluation of differences in mean occlusion and bleeding times. Analysis was also conducted using t-test. Significance was accepted at P<0.05 (two-tailed P value), unless stated otherwise.

## Result

### MALT1 is ubiquitinated in stimulated platelets

**Fig 1:**
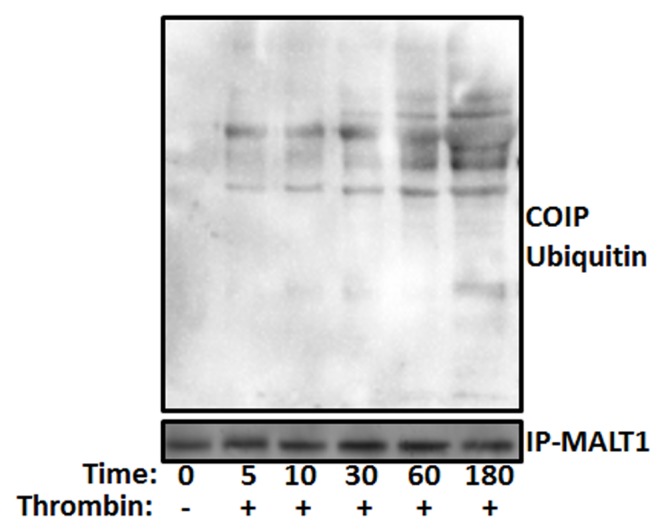
MALT1 is ubiquitinated in thrombin stimulated platelets. Platelets were stimulated with thrombin (0.025 U/mL) for different time points (sec) as indicated in the figure. Platelet lysates were precleared and then incubated with anti-MALT1. Immunoprecipitates were separated by SDS-PAGE and immunoblotted using anti-MALT1 and anti-ubiquitin antibodies.

MALT1 acts not only as a scaffold protein but also as a protease that activates IKK complex in nucleated cells[29], and was shown, in nucleated cell systems such as mast cells, to be heavily polyubiquitinated. To determine whether platelets contain a functional proteasome system, we investigated whether MALT1 is modified by ubiquitin in platelets, under agonist stimulation conditions. Thus, platelets were stimulated with thrombin and MALT1 was immunoprecipitated, under denaturing conditions, and analyzed for its polyubiquitination state, in a time-dependent manner. Indeed, the “modified” MALT1 in the high-molecular-weight fraction was detected by an anti-ubiquitin antibody (Fig. 1), thereby providing evidence that platelets do have a functional proteasome system.

### Platelet aggregation and integrin αIIbβ3 activation is inhibited by proteasome inhibitor

**Fig 2:**
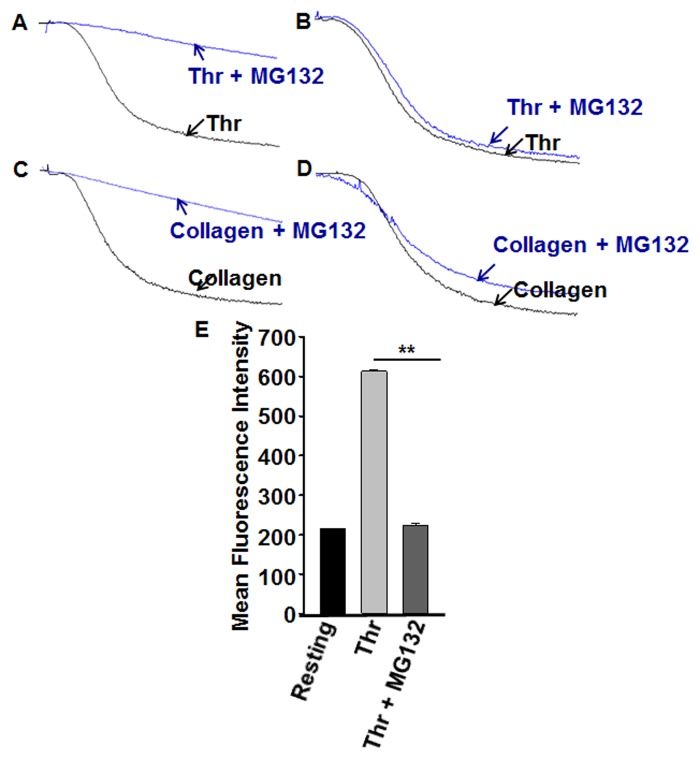
Proteasome inhibitor blocks platelet aggregation and integrin activation. (A–D) Platelets were incubated with proteasome inhibitor (MG132; 10 μM) for 3 min, and platelet aggregation was monitored using aggregometry by stimulating with thrombin ((A) 0.025 or (B) 0.05 U/mL) or collagen ((C) 2.5 or (D) 5 μg/mL) for 3 min. Each experiment was repeated 3 times, with blood obtained from three separate donors. (E) Washed platelets were incubated in the presence or absence of MG132 (10 μM) for 3 minutes and then stimulated with thrombin (0.025 U/mL) for 3 minutes. The reactions were stopped by fixing the platelets with 2% formaldehyde for 30 min at room temperature. Platelets were incubated with FITC-conjugated PAC-1 antibody, the fluorescent intensities were measured by flow cytometry, and the data were plotted as bar diagram); **P < 0.01, Mann-Whitney test.

Platelet hyperactivity is associated with a number of pathophysiological conditions leading to abnormal clot formation resulting in heart attacks and stroke. Given that platelets have a functional proteasome system; our initial experiments were aimed at examining its role in platelet function. Hence, we tested the effect of a proteasome inhibitor (MG132) on platelet activation in agonist dependent manner. It was found that MG132 (10 μM) has the capacity to significantly inhibit platelet aggregation in response to stimulation with 0.025 U/mL thrombin, a relatively low dose (Fig. 2A). Interestingly, 10 μM MG132 produced no effect on thrombin-induced aggregation when we used 0.05 U/mL (Fig. 2B). We also observed substantial blockade of 2.5 μg/mL collagen-triggered platelet aggregation in the presence of MG132 (10 μM; Fig. 2C), whereas it exerted no effect on aggregation when 5 μg/mL of collagen was used (Fig. 2D). These results suggest that the proteasome inhibitor produces inhibitory effects on platelet aggregation only when a low dose of agonist is used. Therefore, in subsequent experiments we employed a thrombin dose of 0.025 U/mL.

It is well documented that integrin αIIbβ3 plays a crucial role in platelet aggregation in response to physiological agonists, and that it mediates thrombus formation[30]. Having established a capacity for the proteasome inhibitor MG132 (10 μM) to block platelet aggregation, we next investigated whether it would be associated with a commensurate inhibition of integrin αIIbβ3 activation. Indeed, our results indicated that pretreating platelets with MG132 (10 μM), results in significant inhibition of 0.025 U/mL thrombin-triggered PAC-1 binding, indicating abrogation of αIIbβ3 activation (Fig. 2E).

### Proteasome inhibitor blocks clot retraction, protects against thrombosis and impairs hemostasis

**Fig 3:**
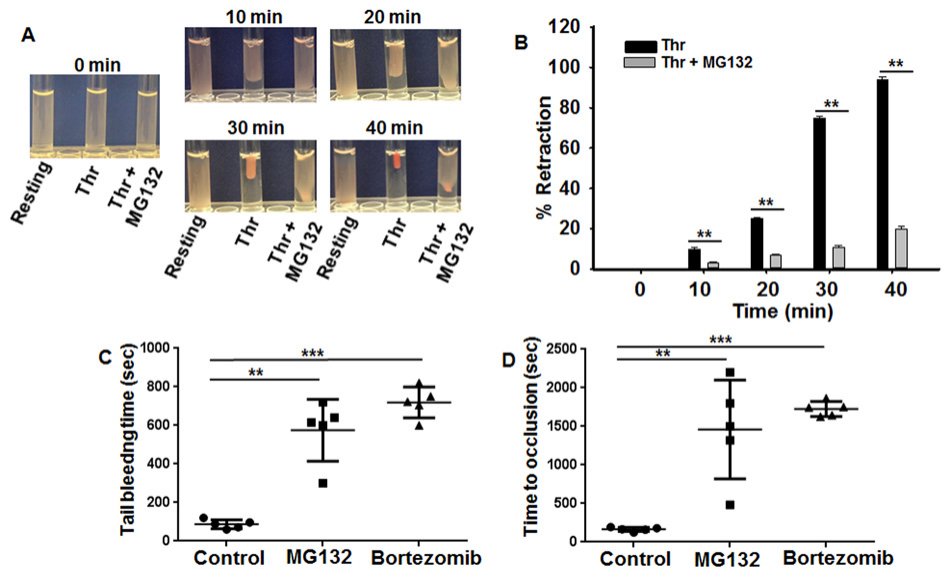
Proteasome inhibitor inhibits fibrin Clot retraction, thrombosis and hemostasis. (A) Platelets were washed and resuspended at 1 × 10^8^/mL in buffer (see “Experimental Procedures”) in the presence of 500 μg/mL human fibrinogen, and fibrin clot was initiated by 0.025 U/mL thrombin at 37°C. Figure represents time frames of a retracting clot at the indicated time points. (B) Clot retraction kinetics curves in the absence or the presence of proteasome inhibitor (MG132). Clot surface areas were assessed by digital processing and plotted as percentage of maximal retraction (*i*.*e*. volume of platelet suspension); **P < 0.01, Mann-Whitney test. (C) Mice were injected either 3% Tween 80 (5 mL/kg, vehicle), 6 mg/kg MG132 (**P < 0.01, Mann-Whitney test) or 2.5 mg/kg bortezomib (***P < 0.001, Mann-Whitney test) in 3% Tween 80 by tail vain injection. (D) One hour post-dosing, FeCl_3_ induced thrombosis measurements were conducted as described in the “Methods” section; **P < 0.01, Mann-Whitney test (for MG132) and ***P < 0.001, Mann-Whitney test (for bortezomib). Each point represents the bleeding time/occlusion time of a single animal (n = 5).

It is notable that many cytoskeletal proteins, other than β-actin, are involved in generating contractile forces in different cell types[31–33]. To investigate the possibility that the proteasome may regulate the ability of platelets to generate contractile forces, we analyzed the effect of MG132 on clot retraction. Remarkably, MG132 (10 μM) significantly delayed clot retraction when compared with the control (Fig. 3A). The extent of retraction was assessed from quantitation of clot surface and is expressed as percentage of total clot curve (Fig. 3B). Given that clot retraction is driven by myosin, a motor protein that interacts with actin, we hypothesized that the observed clot retraction was inhibited in proteasome inhibitor treated platelets due to decreased myosin-mediated force applied to the existing actin cables[34]. Since actin-dependent myosin contractility is thought to contribute to thrombus stability in the arterial system[34], we determined whether proteasome inhibition has any effects on thrombogenesis. Thus, we intravenously injected MG132 (6 mg/kg) or bortezomib (2.5 mg/kg) into C57BL6 mice, and initiated occlusive thrombosis 5 minutes later in a surgically exposed external carotid artery by a brief ectopic application of 7.5% FeCl_3_. This oxidative slur to the vascular wall resulted in the deposition of platelets along the damaged vessel wall that increased over several minutes (Fig. 3C). Typically, complete occlusion of the vessel was found to occur by 2.5 minutes after FeCl_3_ treatment; however, in animals injected with MG132 or bortezomib, occlusion was significantly delayed to more than 28 minutes (Fig. 3C). We next investigated whether the MG132 would exert negative consequences on hemostasis by measuring the tail bleeding time. It was found that mice injected with 6 mg/kg MG132 or bortezomib (2.5 mg/kg) had a significantly prolonged tail bleeding time when compared with control animals (Fig. 3D). Taken together, the above data provide evidence that the MG132 impairs clot retraction, and both MG132 and bortezomib exhibit anti-thrombotic activity, but they do so along with increasing the risk of bleeding.

### Platelet secretion and phosphatidylserine expression is inhibited by proteasome inhibitor

**Fig 4:**
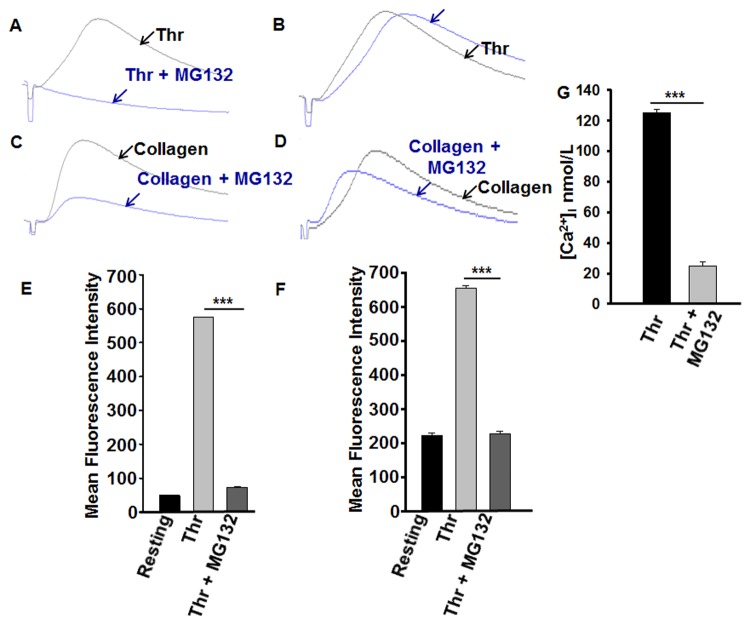
Proteasome inhibitor inhibits platelet dense, alpha granule, PS exposure and release of intracellular calcium. (A–D) Platelets were incubated with proteasome inhibitor (MG132; 10 μM) for 3 min, 12.5 μL of luciferase luciferin was added and stimulated with thrombin (0.025 or 0.05 U/mL) or collagen (2.5 or 5 μg/mL) for 3 min. Release of ATP (for dense granule release) as a luminescence was measured by aggregometer. Each experiment was repeated at least 3 times, with blood obtained from three separate donors. (E) Washed platelets were incubated in the presence or absence of MG132 (10 μM) for 3 minutes and then stimulated with thrombin (0.025 U/mL) for 3 minutes. The reactions were stopped by fixing the platelets with 2% formaldehyde for 30 min at room temperature. Platelets were incubated with FITC-conjugated anti—P-selectin antibody (for alpha granule); ***P < 0.001, Mann-Whitney test and (F) FITC-conjugated Annexin V antibody, the fluorescent intensities were measured by flow cytometry, and the data were plotted as bar diagram; ***P < 0.001, Mann-Whitney test. (G) Platelets were loaded with Fura-2/AM to measure intracellular [Ca^2+^]_i_ in presence or absence of proteasome inhibitor (MG132, 10 μM) upon activation with thrombin (0.025 U/mL). Bar graph representing [Ca^2 +^]_i_ values from platelets treated with or without MG132 (10 μM) in the presence of 7 mM extracellular Ca^2+^, expressed as mean [Ca^2+^]_i_ ± SEM (*n* = 3); ***P < 0.001, Student’s *t*-test.

Platelet secretion is a very important and early event in platelet activation. Thus, we sought to determine whether the proteasome inhibitor (MG132) would exert inhibitory effects on platelet secretion. It was found that MG132 (10 μM) completely inhibited platelet dense granule release in response to 0.025 U/mL thrombin (Fig. 4A), but had not detectable effect when 0.05 U/mL thrombin was used (Fig. 4B). Similarly, MG132 (10 μM) significantly abrogated platelet dense granule release in response to 2.5 μg/mL (Fig. 4C) but not 5 μg/mL of collagen (Fig. 4D).

We next examined the role of the proteasome in alpha granule secretion, by measuring the marker P-selectin using flow cytometry. Indeed, it was found that MG132 (10 μM) has the capacity to inhibit P-selectin expression in (0.025 U/mL) thrombin-stimulated platelets, compared to the control (Fig. 4E). Together, these data show that proteasome inhibitor MG132 blocks platelet dense and alpha granule release.

It is well known that the “latter” platelets are characteristically elevated in cytosolic Ca^2+^ with attached microparticles, with the negatively charged lipid phosphatidylserine (PS)[35] exposed. Therefore, we examined if the proteasome inhibitor MG132 inhibits phosphatidylserine expression in thrombin-stimulated platelets. Our results revealed that pretreatment of platelets with proteasome inhibitor (MG132; 10 μM) significantly inhibited 0.025 U/mL thrombin-induced PS expression (Fig. 4F), indicating that the proteasome regulates PS exposure.

### Proteasome inhibitor blocks elevation in intracellular calcium in platelets

Since platelet function (e.g., aggregation and secretion) critically depend on increases in cytosolic calcium concentration ([Ca^2+^]_i_)[36], we examined the effect of the proteasome inhibitor MG132 on [Ca^2+^]_i_. It was found that platelets that are treated with 10 μM of the proteasome inhibitor MG132 had a significantly lower [Ca^2+^]_i_ in response to thrombin (0.025 U/mL (Fig. 4G), when compared with that of the control (Fig. 4G).

### The CBM signaling complex is present in platelets, and is upstream of IKKβ

**Fig 5:**
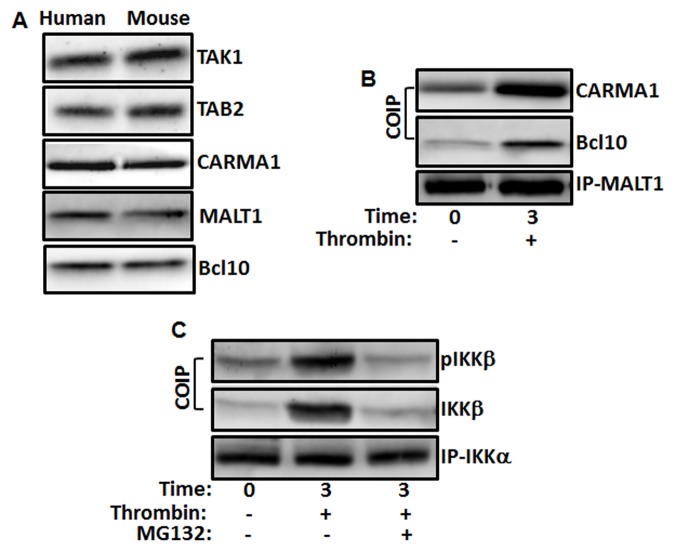
TAK1, TAB2 and CBM proteins are present in platelets. (A) Human and Mouse platelets were held resting and extracts from them were subjected to immunoblotting with anti-CARMA1, anti-MALT1, anti-Bcl10, anti-TAK1 and anti-TAB2 antibodies. (B) CBM complex is present in stimulated platelets. Human platelets were stimulated with thrombin (0.025 U/mL) for 3 min. Platelet lysates were precleared and then incubated with anti-MALT1. Immunoprecipitates were separated by SDS-PAGE and immunoblotted using antibodies to MALT1, Bcl10 and CARMA1. (C) Association of IKKα, IKKβ and pIKKβ is inhibited by proteasome inhibitor in stimulated platelets. Platelets were treated with MG132 (10 μM) for 3 min and stimulated with thrombin (0.025 U/mL) for 3 min. Platelet lysates were precleared and then incubated with anti-IKKα. Immunoprecipitates were separated by SDS-PAGE and immunoblotted using antibodies to IKKα, IKKβ and pIKKβ.

In our earlier work, we have shown that IKKβ plays an important role in the regulation of platelet SNARE machinery[13]. Thus, we sought to delineate the signaling molecules upstream of IKK. As can be seen in Fig. 5A, it was found that CARMA1, Bcl10 and MALT1 (i.e., the CBM complex), along with its upstream kinases TAK1 and TAB2, are present in platelets. Hence, we investigated the CBM complex formation in stimulated platelets, by employing a MALT1 immunoprecipitation-based strategy. Platelets were stimulated with thrombin (0.025 U/mL), MALT1 was immunoprecipitated, and we found that Bcl10 and CARMA1 are associated with MALT1 in stimulated platelets, but not in comparison with unstimulated platelets (Fig. 5B). These findings suggest that stimulated platelets form functional CBM complex, and that it may regulate the activation of IKK.

To determine whether MALT1 ubiquitination is a primary event in response to platelet activation, we determined the onset of MALT1 ubiquitination. Indeed, as shown in Fig. 1, we observed that MALT1 is polyubiquitinated in thrombin stimulated platelets. Furthermore, we found that IKKβ is phosphorylated, and in turn phosphorylates and associates with IKKα, in thrombin-stimulated platelets (Fig. 5C). However, when platelets were pretreated with proteasome inhibitor (MG132; 10 μM) and stimulated with thrombin, we observed that phosphorylation of IKKβ is abrogated, and it is dissociated from IKKα. This suggests that the onset of MALT1 ubiquitination precedes IKK activation.

### IKKγ, TAK1 and TAB2 associate with ubiquitin-conjugated MALT1

**Fig 6:**
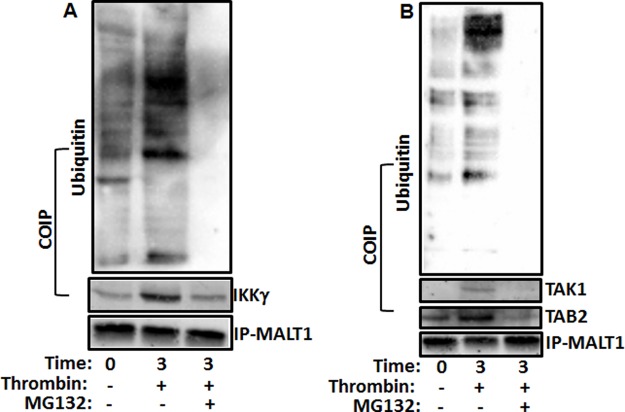
IKKγ, TAK1 and TAB2 associate with ubiquitin-conjugated MALT1. (A) Platelets were treated with MG132 (10 μM) for 3 min and stimulated with thrombin (0.025 U/mL) for 3 min. Platelet lysates were precleared and then incubated with anti-MALT1. Immunoprecipitates were separated by SDS-PAGE and immunoblotted using antibodies to MALT1, IKKγ and ubiquitin. (B) Platelets were treated with MG132 (10 μM) for 3 min and stimulated with thrombin (0.025 U/mL) for 3 min. Platelet lysates were precleared and then incubated with anti-MALT1. Immunoprecipitates were separated by SDS-PAGE and immunoblotted using antibodies to MALT1, TAK1, TAB2 and ubiquitin.

IKKγ was recently shown to contain a ubiquitin binding domain (UBD)[37–39], so we asked whether IKKγ can associate with the ubiquitinated form of MALT1, in response to platelet activation. Indeed, we were able to detect (co-immunoprecipitate) ubiquitin-conjugated MALT1 in association with IKKγ, in thrombin-activated platelets (Fig. 6A). Conversely, when platelets were pretreated with proteasome inhibitor (MG132; 10 μM) and stimulated with thrombin, IKKγ association with MALT1 was inhibited, and MALT1 ubiquitinated was blocked (Fig. 6A). These data indicate that IKKγ associates only with the ubiquitinated form of MALT1, in stimulated platelets.

In nucleated cells, it has been shown that transforming growth factor beta-activated kinase 1 (*TAK1; kinase) and the TAK1 binding protein 2 (TAB2; adaptor protein) are upstream and regulate CBM complex formation*. So, we next determined if TAK1 and TAB2 associate with ubiquitin chains of MALT1 in stimulated platelets using co-IP experiments. It was found that both proteins are in fact recruited to ubiquitinated MALT1, upon thrombin stimulation of platelets (Fig. 6B). Furthermore, the proteasome inhibitor (MG132; 10 μM) blocked the association of TAK1 and TAB2 with MALT1, and inhibited its ubiquitination as well, in stimulated platelets (Fig. 6B). This finding suggests that TAK1 and TAB2 are recruited upon ubiquitination of MALT1, in activated platelets.

### SNARE complex formation is disrupted by proteasome inhibitor

**Fig 7:**
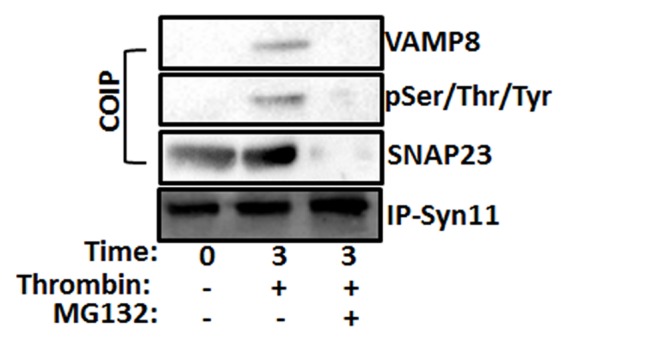
Proteasome inhibitor inhibits SNARE complex formation. Human platelets were incubated with proteasome inhibitor (MG132, 10 μM) and stimulated with thrombin (0.025 U/mL, 3 minutes). Platelet lysates were precleared and then incubated with anti-syntaxin-11. Immunoprecipitates were separated by SDS-PAGE and immunoblotted using antibodies to syntaxin-11, SNAP-23, phosho-Ser/Thr/Tyr (pSer/Thr/Tyr), and VAMP-8 as indicated.

In earlier studies Karim *et al*. (2013)[13] showed that IKKβ regulates SNARE assembly in stimulated platelets. The present work documented the presence of a functional proteasome in platelets, and that MALT1 is ubiquitinated and regulates IKKβ activation, in these anucleated cells (Fig. 1). We also observed that a proteasome inhibitor (MG132; 10 μM) has the capacity to inhibit platelet dense and alpha granule release (Fig. 4A-E). As a logical extension to our earlier studies[13], we sought to investigate whether the proteasome may also regulate platelet SNARE assembly. Thus, platelets were treated with proteasome inhibitor (MG132; 10 μM), stimulated with thrombin, before we immunoprecipitated using an antibody against Syn-11, and detected SNAP-23, phospho-SNAP-23 and VAMP-8. Indeed, we were first able to demonstrate SNARE complex formation in stimulated platelets (Fig. 7), as we previously showed[13], and found that proteasome inhibitor blocked its formation, in stimulated platelets (Fig. 7). These data indicate that the platelet proteasome regulates SNARE assembly, and cargo release form the dense and alpha granule.

## Discussion

Recently, we have shown that IKK is central in controlling membrane fusion[13], and that its activation is required for regulating SNARE machinery[13] in platelets. Here, we show that platelets have a functional proteasome which controls activation of IKK and membrane fusion. To this end, while the recruitment of Bcl10/MALT1 to CARMA1 (CBM complex) was found to be essential for IKK activation in T cells, the molecular mechanisms of IKK activation upstream of CBM have not been completely elucidated, in platelets. In our present study, we present the first evidence that ubiquitination of MALT1 functionally links CBM and IKK complexes. Thus, firstly, using a pharmacological inhibitor, we show that the proteasome plays a vital role in platelet functional responses, such as aggregation, αIIbβ3 activation, dense and alpha granule release, PS exposure, and regulation of intracellular calcium levels. Secondly, we found that the proteasome inhibitor has the capacity to delay thrombus formation *in vivo* and prolong tail bleeding time, indicating a function for the proteasome in these processes. Thirdly, we also observed that MALT1 ubiquitination coincides with the activation of the IKK/NF-κB-signaling pathway, in platelets. Finally, we observed that the proteasome inhibitor blocks CBM complex formation, and abrogates the interaction of IKKγ and MALT1 as well as SNARE formation. This is important given that IKKγ is critical for NF-κB signaling, in platelets. Collectively, these data demonstrate that polyubiquitination of MALT1 is essential for directing platelet signaling to the NF-κB pathway, and underscore the proteasome system as a potential target for the therapeutic management of thrombogenesis.

Platelets do contain a functional proteasome that modifies cellular proteins to mark them as proteasome substrates. In fact, the proteome of quiescent platelets contains numerous ubiquitin-protein conjugates whose adduction was increased on stimulation, which is consistent with the previous studies showing that collagen activation stimulates ubiquitination of platelet Syk kinase[40] through the E3 ligase Cbl-b[41]. Moreover, although CARMA1 and Bcl10 are involved in ubiquitination of IKKγ, both proteins are dispensable for TAK1-dependent IKKα/β phosphorylation[42]. Mechanistically, it is unclear how these separate pathways are integrated. Here, we report that the critical components, including CARMA1, Bcl10, MALT1, TAB2/TAK1 and IKKγ are not only present in platelets, but they also associate. While it is not clear whether the aforementioned molecules associate directly or indirectly, these findings suggest that these signaling molecules are acting in concert to regulate platelet function. However, we cannot exclude the possibility that some of these signaling components (e.g. TAK1) can perform certain tasks independent of the other mediators. And future studies will determine how TRAF6 is involved in the regulation of CBM complex formation and regulation, in platelets.

MALT1 has been found to polyubiquitinated, in both Mast and T-cells[23]. Polyubiquitination is detectable relatively soon after stimulation and controls the scaffolding function of MALT1. Indeed, this modification has been attributed to the E3 ligase TRAF6, and is believed to enable the recruitment of IKKγ, and thereby the activation of NF-κB, in these cells[23]. Recent studies demonstrated a conserved function for Bcl10-MALT1 in directing G-protein-coupled receptors (GPCR) to NF-κB activation[43–46]. The CARMA1 homologue CARMA3 (CARD10) functions as a scaffold for GPCR-initiated activation, which is abrogated by TRAF6 deficiency[47]. Thus, ubiquitination of the C-terminus of MALT1 may be relevant for activation of NF-κB in various physiological and pathological settings. Importantly, we establish for the first time that platelets, like nucleated cells, express a functional proteasome that enables them to ubiquitinate their proteome. This “decoration” increases on stimulation and modulates an array of responses from several receptors that engage the SNARE machinery and ultimately release their cargo. Furthermore, we observed that inhibition of ubiquitination of MALT1 inhibits SNARE assembly, suggesting that ubiquitination of MALT1 regulates activation of IKK in platelets. We also provided evidence that polyubiquitination of MALT1 controls CBM complex formation, and activation of IKKβ in platelets. The importance of this finding derives, in part, from our recent work in which we have shown that IKK regulates SNARE assembly and membrane fusion[13]. Finally, the platelet ubiquitin system modulates their response to thrombotic stimuli, and proteasome inhibition effectively delayed arterial thrombogenesis. These findings suggest that targeting the proteasome to ameliorate polyubiqutination of MALT1 may be a viable target for the development of new antithrombotic drugs.
